# Antibacterial Properties of PCL@45s5 Composite Biomaterial Scaffolds Based on Additive Manufacturing

**DOI:** 10.3390/polym16233379

**Published:** 2024-11-30

**Authors:** Chen Zhang, Yixian Ru, Jinchao You, Runyi Lin, Shihao Chen, Yi Qi, Dejing Li, Cheng Zhang, Zhenli Qiu

**Affiliations:** 1 School of Materials and Chemistry Engineering, Minjiang University, Fuzhou 350108, China; 15375845279@163.com (J.Y.); 13163893586@163.com (R.L.); lidejing@mju.edu.cn (D.L.); zhangcheng@mju.edu.cn (C.Z.); 2 School of Pharmaceutical Sciences, Jilin University, Changchun 130021, China; 15617361333@163.com (Y.R.); shchen21@mails.jlu.edu.cn (S.C.); qiyi23@mails.jlu.edu.cn (Y.Q.)

**Keywords:** three-dimensional printing, composite scaffolds, PCL, 45s5, antibacterial properties

## Abstract

This study focuses on the development of polymer–bioglass composite bone scaffolds for the treatment of bone defects. PCL particles and 45s5 bioglass powder were employed as raw materials to fabricate PCL/45s5 composite wires with mass fractions of 5 wt%, 10 wt%, and 20 wt% via the twin-screw extrusion method. A cylindrical porous model was established using 3D modeling software, and a porous composite scaffold was constructed through the melt deposition manufacturing process. The macroscopical characterization of composite stock and composite powder was analyzed. The melt flow rate, water contact angle, elastic modulus, in vitro degradation rate, and antibacterial property of composite scaffold were measured. The experimental results showed that the incorporation of 45s5 bioglass into PCL material gave the composite better antibacterial properties, effectively reduced the flow rate of the material, increased the hydrophobicity of the material, and improved the rigidity and biocompatibility of the PCL material. This study offers initial insights into the use of synthetic bone tissue engineering scaffolds for clinical bone repair treatments.

## 1. Introduction

Bone defects are caused by trauma or surgery [1]. Bone defects often lead to nonunion, delayed or non-healing, and local functional impairment. In the past few decades, the aging population has intensified, and bone tumor resection, infection, trauma, etc., often lead to bone defects. Bone tissue engineering has become a key research topic in the field of regenerative medicine [2]. Alzhavan et al. synthesized graphene nanoribbons deposited on polydimethylsiloxane and applied them to selective two-dimensional templates to accelerate the proliferation and differentiation of human bone marrow mesenchymal stem cells. Other scholars have developed a new type of core–shell microcapsule with stem cell core and biomass shell by using the aqueous phase microfluidic electric spray technology; animal experiments show that the core–shell microcapsule with stem cells has good biocompatibility and therapeutic effect on bone defects [3,4]. Autologous bone grafting is the favored option for treating bone defects in clinical settings [5]. However, autologous bone grafting influences difficulty in obtaining, inability to change shape as needed, and increased risk of infection in patients due to secondary surgery, which makes its clinical application difficult [6]. Traditional bone implants such as allograft bone and metal also have a series of problems, including stress shielding risk [7], potential immunogenicity hazards, and high incidence rate in donor sites [8], making it difficult to meet clinical needs.

In traditional bone repair materials, metal materials such as titanium and titanium alloys are often used as materials for bone implants. Titanium and titanium alloys have excellent mechanical properties, good biocompatibility, and corrosion resistance and are considered to be one of the most compatible metals in the human body [9,10,11]. However, titanium alloys still exhibit a “stress shielding” effect after being implanted in the human body [12], with bacterial infection, low strength, and poor surface wear resistance. Although some researchers have modified titanium materials for antibacterial purposes [13,14], the aluminum and vanadium elements present in traditional titanium alloys, such as Ti-6Al-4V, may still pose potential risks to human health [15]. Polymer materials are widely used in the biomedical field, and the commonly used polyester material polycaprolactone (PCL) has good biocompatibility, degradability, and non-toxicity [16]. However, as a bone repair material, it may still exhibit different reactions from expectations after being implanted in the human body [17], and its mechanical strength and toughness may not meet the requirements [18]. Moreover, PCL itself does not have osteogenic activity, and during degradation, it releases H^+^, which can reduce the pH of the implant site and cause inflammation [19,20]. Bioceramic materials are widely used in the biomedical field and have many advantages, but there are also some drawbacks [21], such as bioceramics not having sufficient mechanical strength and toughness to bear weight and the high melting point of the material, making it difficult to form [22,23]. However, with the development of additive manufacturing technology, ceramic materials have been widely used in biomedicine, aerospace, and other fields. Studies have shown that the fusion of PCL and bioceramic materials can enhance the mechanical strength and toughness of the composite material, making its properties more stable compared to single materials [24]. Bioceramics gradually release Ca^2+^ and PO_4_^3−^ during degradation to promote bone construction and create an alkaline environment that can neutralize the H^+^ released by polyester degradation, reducing the probability of inflammation. Therefore, polyester/bioceramic composite materials can better promote bone repair [25]. Among them, 45s5, as a bioceramic material, not only promotes bone repair but also exhibits strong antibacterial effects. Studies have shown that the release of soluble sodium and calcium particles in aqueous solution by 45s5 leads to an increase in pH value, and the fragmented structure of 45s5 particles themselves can damage bacterial cell walls, which are important reasons for the antibacterial effect of this material [26]. Adjusting the ratio of components in the composite material and making full use of additive manufacturing technology can produce a bone repair scaffold that meets expectations. Prior to this, there have been reports on antibacterial scaffolds for bone tissue regeneration. In addition to composite antibacterial materials, antibacterial properties can also be improved by modifying the scaffold with antibacterial coatings [27,28].

Additive manufacturing technology, also known as 3D printing technology, uses digital modeling to print and construct the required objects layer by layer using bondable materials. Additive manufacturing technology is widely used in manufacturing, healthcare, aerospace, and other fields [29,30]. The additive manufacturing technologies used in the construction of bone implants mainly include (FDM) [31], electron beam freeform fabrication (EBF) [32], selective laser sintering (SLS) [33], and stereolithography (SLA) [34]. The porous scaffold structure obtained by additive manufacturing technology printing is closer to natural bone, and the porous structure with a pore size of 100–1000 μm is more suitable for nutrient exchange and has better effects on promoting vascular formation and bone formation [35,36,37]. Additive manufacturing technology is the most promising manufacturing method in the fabrication of bone scaffolds with porous structures, and the use of additive manufacturing technology for the personalized fabrication of bone implants contributes to patient care, significantly reduces the time required for bone implant preparation, reduces material waste, and allows for the design and creation of more complex structures [38,39].

In previous studies, Tavakoli, Chenglong Wang, and others conducted experiments on the mechanical properties and biocompatibility of PCL@bioglass scaffolds and PCL@bioglass@gel scaffolds, validating their findings through animal and cell experiments. However, they lacked evaluations of the thermodynamic properties and antibacterial activity of PCL@bioglass materials [40,41]. Based on this, we conducted relevant research. This study designs a three-dimensional model using fused deposition modeling technology; selects PCL material and 45s5 to prepare PCL/45s5 composite scaffolds with mass fractions of 5 wt%, 10 wt%, and 20 wt% 45s5; observes their characterization; measures their physical properties such as contact angle and elastic modulus; and analyzes their antibacterial efficacy, providing preliminary research for the clinical application of artificially synthesized bone tissue engineering scaffold materials for bone repair.

## 2. Materials and Methods

### 2.1. Fabrication of 3D-Printed PCL, PCL/45s5 Scaffolds

**Figure 1:**
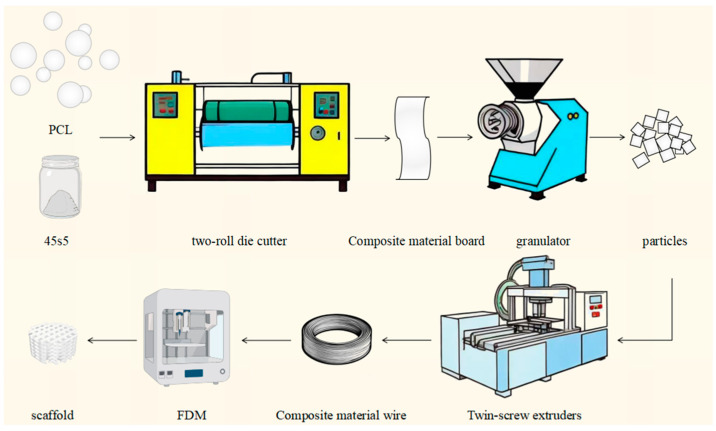
The scheme of preparation of the scaffolds.

PCL purchased from SOLVAY (Brussels, Belgium) was mixed with 45S5 bioactive glass purchased from Kunshan Technology New Materials Co., Ltd. (Kunshan, China) in different mass fractions (5%, 10%, and 20%), with a particle size range of 0–80 μm. These synthetic materials constitute three parallel experimental groups, labeled as PCL/45s5 5 wt%, PCL/45s5 10 wt%, and PCL/45s5 20 wt%, each representing a distinct quality score for 45s5. PCL was used as a reference group. Prior to mixing, an air blast oven (XMTD-8222, Shanghai Jinghong Experimental Instrument Co., Ltd., Shanghai, China) was used to dry 200 mesh PCL particles (Capa 6500; SOLVAY, Alorton, IL, USA) for 8 h at 40 °C. After evenly mixing the composite material, it was cut into rectangular strips of 100 mm by 40 mm by 2 mm using a two-roll die cutter (LN-LT-4, Guangdong Lina Industrial Co., Ltd., Dongguan, China) set to 45 °C. The granulation process was then carried out using a granulator (JD1A-90, Shanghai Delixi Switch Co., Ltd., Shanghai, China). The produced composite particles were prepared into strands suitable for 3D printing composite scaffold using twin-screw extruders (PloyLab Os, Thermo Fisher Scientific, Bremen, Germany). The freshly made wire is uneven and includes burrs, which makes it unsuitable for 3D printing scaffolds because 45s5 bioactive glass and PCL have different physical characteristics. To fix this issue, you must use a granulator to chop the strand into composite particles once more until the wire is 1.75 mm thick and free of surface burrs. Next, Solidworks 2022 was used to design the cylindrical support model, and Snapworks Luban4.7.2 was used to design the process parameters. The bottom diameter of the support was 15 mm, the height was 4 mm, the porosity was 50 wt%, the brush height was 0.28 mm, the room temperature was 20 °C, and the nozzle temperature was 80 °C. The hot bed temperature is 45 °C. In the end, FDM technology was used to fabricate four sets of scaffolds made from both composite substances and PCL (as shown in Figure 1).

### 2.2. Macroscopic Observation of the Scaffolds

From the three experimental groups (PCL/45s5 5 wt%, PCL/45s5 10 wt%, and PCL/45s5 20 wt%) and a control group made up of only PCL, a composite scaffold is chosen, resulting in a total of 12 scaffolds. The scaffolds’ diameter and height were measured separately with a vernier caliper (Shanghai Tool Factory Co., Ltd., Shanghai, China), and using a precision balance, they were weighed (Kunshan Scientific Instrument Co., Ltd., Kunshan, China). Average values were computed from the gathered data.

### 2.3. Microscopic Morphology of 45s5 Powder

To examine the microstructural features of the composite scaffold, SEM (E-1045, HITACHI; Tokyo, Japan) captured images of the PCL/45s5 scaffold and 45s5 bioglass powder, respectively, at magnifications of 1000×, 2000×, 5000×, and 10,000×.

### 2.4. XRD Analysis

Firstly, PCL composite materials containing 5 wt%, 10 wt%, and 20 wt% 45s5 bioglass were compressed into samples with a diameter of 20 mm and a thickness of 1 mm, followed by X-ray diffraction analysis using an X-ray diffractometer. The current was set at 15 mA and the voltage at 40 kV. At a rate of four times per minute, we scanned the 2θ range between 5° and 40°. Following the experiment, we processed and analyzed the records using MDI Jade 6 before producing a chart.

### 2.5. Melt Flow Rate

After mixing 45s5 bioglass and PCL with a twin screw, the composite material and PCL material were cut into particles. Let the particle samples be divided into four categories: PCL, PCL/45s5 5 wt%, PCL/45s5 10 wt%, and PCL/45s5 20 wt%. To assess the melt index of composite materials with varying proportions, each sample was heated to 125 °C. In accordance with ASTM D1238, the melt flow rate was then ascertained using a melt flow rate tester (XNR-400A; Chengde Dajia Instrument Co., Ltd., Heibei, China).

### 2.6. Heating Rheological Test

Initially, the samples were placed in a flat vulcanizing machine and compressed to 20 mm in diameter and 1 mm in thickness. Next, we categorized the samples into four groups: pure PCL, PCL/45s5 at 5 wt%, PCL/45s5 at 10 wt%, and PCL/45s5 at 20 wt%. To analyze the changes in viscosity of the composite materials during heating, the materials were heated from 75 °C to 120 °C and then evaluated using a polymer rotating rheometer system (DHR-2, TA Instruments; Newcastle, DE, USA).

### 2.7. Differential Scanning Calorimetry (DSC)

Four groups were also created from the particle samples. The composite material is reduced from room temperature to −80 °C at the same speed, then raised to 100 °C to eliminate thermal history, cooled down again to −80 °C, and the performance changes of the material between −80 °C and 100 °C are tested using differential scanning calorimetry.

### 2.8. Contact Angle

One stent is randomly selected from four groups of pure PCL, PCL/45s5 5 wt%, PCL/45s5 10 wt%, and PCL/45s5 20 wt%, and its contact angle is measured using a water contact angle measuring instrument (JC2000D, Shanghai Zhongchen Digital Technology Equipment Co., Ltd., Shanghai, China).

### 2.9. Modulus of Elasticity

Three supports are taken from each of the four groups of samples, and their elastic modulus is tested using a universal material testing machine (AG-X plus 100 KN; SHIMADZU, Shanghai, China); we kept a record of the tension and stress.

### 2.10. In Vitro Degradation Rate

Randomly select scaffolds as experimental group PCL/45s5 5 wt%, PCL/45s5 10 wt%, PCL/45s5 20 wt%, and control group (*n* = 4). After recording the quality, the scaffold is placed in a sterilized plastic cup, 10 mL of 75% alcohol solution is added for ultrasonic disinfection for 30 min, and then it is placed on an ultra-clean table to air dry. The scaffold is placed in a 50 mL cell culture bottle and immersed in 30 mL PBS-simulated body fluid. The culture area is 25 cm^2^, incubate at 37 °C, observe for 30 days, remove the scaffold, and dry it in a 40 °C air drying oven to a constant mass. Record the quality changes in the scaffold after drying.

Δ(wt%) = [(W_0_ − W_t_)/W_0_] × 100% (1)

In the formula, ∆ represents the in vitro degradation rate, W_0_ represents the mass of each scaffold before degradation, and W_t_ represents the weight of the scaffold after incubation and degradation in simulated body fluid for 30 days.

### 2.11. Antibacterial Test

#### 2.11.1. Preparation of Medium

To prepare 1000 mL of liquid LB medium, 1000 mL of sterile water is added, along with 10 g of sodium chloride, 5 g of yeast extract, and 10 g of peptone. The pH is adjusted to 7 using solid sodium hydroxide, and the mixture is then sterilized in a high-temperature sterilizer for 40 min. Additionally, for the preparation of 1000 mL of LB solid medium, 20 g of agar powder is added to another 1000 mL of sterile water. The pH is adjusted to 7 with solid sodium hydroxide, and the solution is sterilized in a high-temperature sterilization pot. Finally, it is poured into plates to cool and solidify, resulting in the LB solid medium plates.

#### 2.11.2. Antibacterial Circle Test

A total of 10 μL of the bacteria (*Escherichia coli* and *Staphylococcus aureus*, Aoqi Kehua medical supply chain management service Co., Ltd., Tianjin, China) was extracted from the glycerol storage solution containing the target bacteria using a pipette gun and was placed into 50 mL of liquid LB medium for overnight culture at 37 °C, 220 r/min. On the next day, 10–20 μL bacteria cultured overnight were poured into the LB plate on a super-clean table, and the plate was evenly coated with a coating rod. Four sets of scaffolds, pure PCL, PCL/45s5 5 wt%, PCL/45s5 10 wt%, and PCL/45s5 20 wt%, were placed in the center of the well-coated LB plate and placed in an incubator at 37 °C. After 1 day of cultivation, we then observed whether there was a bacteriostatic zone around the scaffold and observed and measured the diameter and clarity of the bacteriostatic zone.

#### 2.11.3. Gram Staining Test

Colonies are picked from the solid culture medium and placed in 20 mL of liquid culture medium for overnight cultivation. When the colonies grow to a certain density, take a portion of the bacteria to prepare a suspension and dilute it with physiological saline to the appropriate concentration. A total of 10 μL of the prepared bacterial suspension is taken with a pipette and applied onto a glass slide. The slide is heated with an alcohol lamp to allow the bacteria to adhere to it and be fixed. Moreover, 1–2 drops of purple staining agent are drawn onto the fixed plaque on the glass slide using a dropper. The staining is carried out for 30–60 s, then distilled water is drawn, and the residual staining solution is rinsed off with slow water flow. Next, 1–2 drops of iodine solution are added and allowed to stand for 30–60 s. The residual iodine solution is rinsed off with distilled water, followed by washing and decolorizing with alcohol, and the residual alcohol is rinsed off with distilled water. Finally, 1–2 drops of magenta staining agent are added and allowed to stand for 30–60 s before being rinsed with distilled water. The glass piece is dried and can then be used to observe the staining of bacteria.

## 3. Results

### 3.1. Morphology Observations

**Figure 2:**
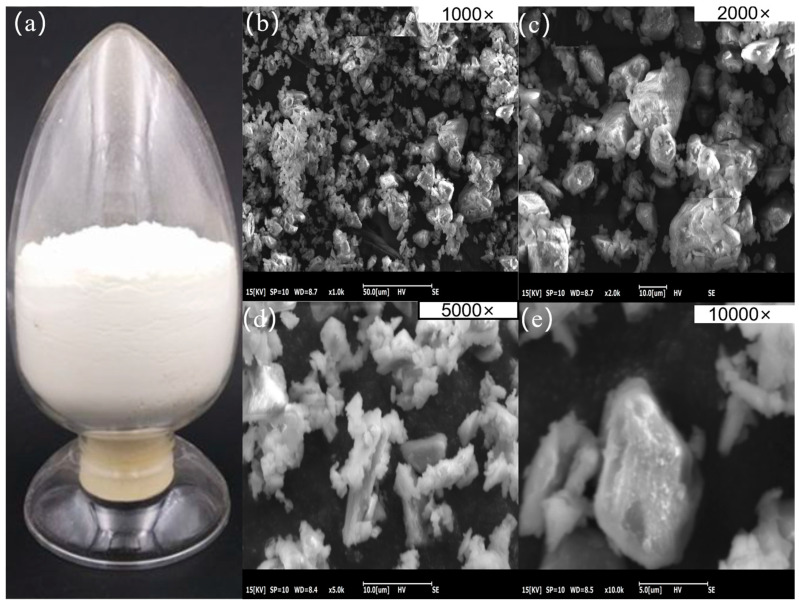
Macroscopic state of 45s5 powder in glass container and SEM image. (**a**) macroscopic characterization; (**b**) 1000× scanning electron microscope; (**c**) 2000× scanning electron microscope; (**d**) 5000× scanning electron microscope; (**e**) 10,000× scanning electron microscope.

At room temperature, 45s5 bioactive glass looks like a fine, white, and even powder when kept in a glass container. It does not have any noticeable crystal structures or obvious defects (as shown in Figure 2a). The microstructure of the 45s5 powder may be seen using scanning electron microscopy (SEM) at 1000×, 2000×, 5000×, and 10,000× magnifications (as shown in Figure 2b–e). The powder particles exhibit irregular polyhedral crystalline shapes, and at a magnification of 10,000×, individual crystals can be clearly observed, with particle sizes typically ranging from 0.1 to 5 μm.

**Figure 3:**
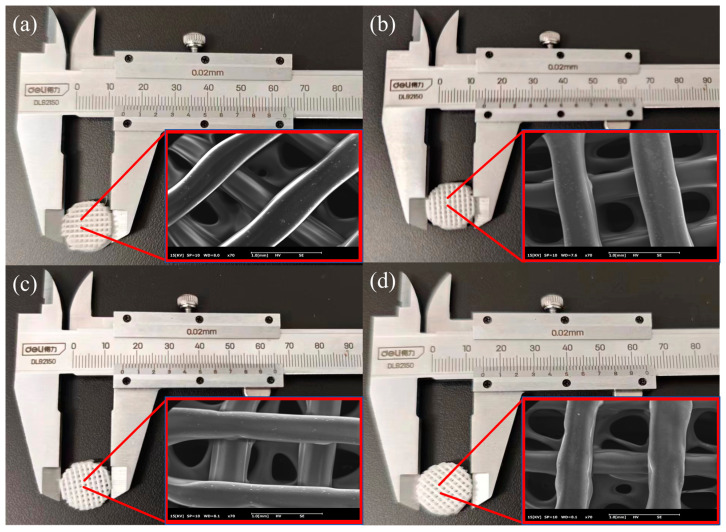
Macroscopic morphology and SEM of composite artificial bone scaffold. (**a**) pure PCL scaffolds; (**b**) PCL/45s5 5 wt% scaffold; (**c**) PCL/45s5 10 wt% scaffold; (**d**) PCL/45s5 20 wt% scaffold.

Excessive or insufficient pore size is not conducive to cell adhesion. The porosity of artificial bone scaffolds, which ranges from 30% to 90%, is comparable to that of human bone trabeculae. The porosity of artificial bone scaffolds is considered the best [26]. In order to facilitate cell adhesion, the porosity of the scaffold is 50 wt%. Figure 3 shows that the PCL/45s5 composite bone scaffold has a cylindrical shape formed by stacking filaments of the composite material through FDM printing, creating a porous structure. The scaffold has a radius of approximately 7.5 mm and a height of about 4 mm. The color of the scaffolds in different groups varies due to the differing concentrations of 45s5 bio-glass. The higher the concentration, the darker the color of the composite scaffolds. The data for the composite scaffolds is listed in Table 1. Every measurement item in the group has a standard deviation of less than 0.5, indicating that the weight, diameter, and height differences of the PCL bone scaffold based on 3D printing are small and demonstrate excellent repeatability in scaffold manufacturing.

The scanning electron microscope image of the scaffold structure shows the staggered pore structure of the scaffold. Consistent porosity is crucial for promoting inward cell growth. The staggered printed linear arrangement of bone scaffolds can effectively maintain a consistent pore structure, providing effective growth space for inward cell growth and meeting the biological requirements of bone regeneration.

### 3.2. X-Ray Diffraction (XRD) Analysis

**Figure 4:**
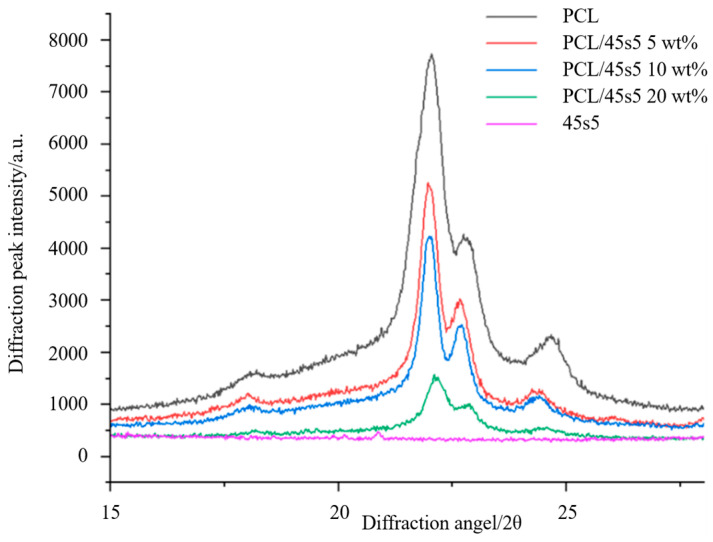
XRD pattern of the samples.

It is well known that 45s5 bio-glass is amorphous, while PCL is semicrystalline. From Figure 4, we can observe the characteristic peaks of PCL at 2θ = 21.9° and 2θ = 24.2° [42]. Additionally, the peak of PCL shifts to the right, which we believe may be due to a phase transition during the preparation of the composite material. In contrast, 45s5 does not show any peaks. In the mixed materials, the composites with different mass fractions of 45s5 display the same characteristic peaks as PCL without any new peaks appearing. This suggests that no new crystalline phases are formed in the mixed materials.

### 3.3. Melt Flow Rate of Composite Materials

**Figure 5:**
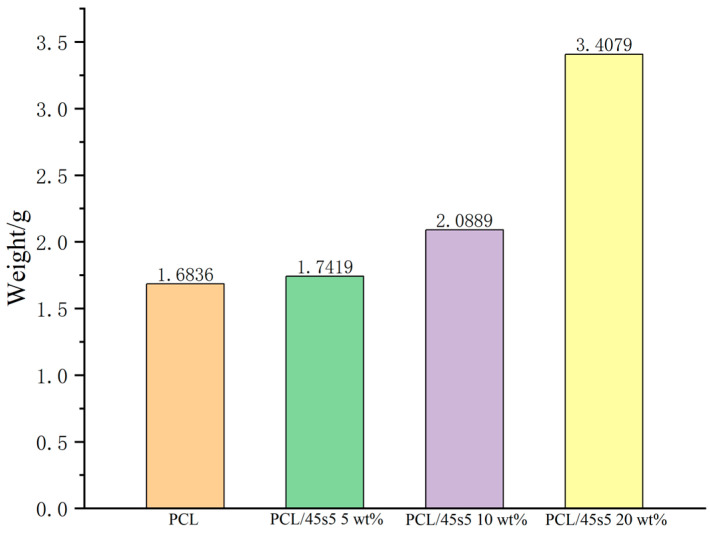
Histogram of melt flow rate.

Figure 5 shows the melt flow rate of PCL/45s5 composite materials containing 45s5 bioglass with different mass fractions. In composite materials, the higher the mass fraction of 45s5 bioglass, the higher its fluidity. This characteristic provides convenience for the preparation process of composite materials and is also more conducive to additive manufacturing.

### 3.4. DSC Result of Composite Materials

**Figure 6:**
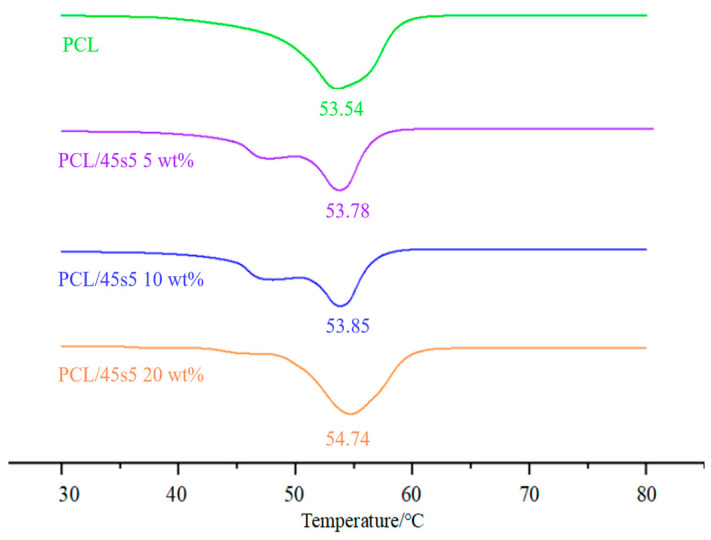
DSC curves of PCL/45s5 composites with different 45s5 content.

Figure 6 shows the DSC curve obtained after eliminating thermal history from −80 °C to 100 °C. From the DSC curve of the composite materials in Figure 6, we explore the relationship between the melting point of the PCL/45s5 composite and the content of 45s5 bio-glass. It can be observed from the graph that as the content of 45s5 increases, the melting point of the composite material also increases, from 53.54 °C for pure PCL to 54.74 °C for a 20 wt% PCL/45s5 composite. This phenomenon may be due to the formation of a specific molecular arrangement between PCL and 45s5 bioglass upon mixing. Additionally, we observed a shoulder peak before the melting peak of the composite material. We suggest that this shoulder peak may be related to phase separation occurring in the composite material at this temperature, particularly at lower 45s5 contents. This phase separation leads to different thermal characteristics in different regions of the composite, and the shoulder peak might reflect local melting or phase transitions occurring in certain areas of the material.

### 3.5. Contact Angles

To examine how the addition of 45s5 bioglass affects the hydrophilicity of bone scaffolds, we conducted hydrophilicity tests on the composite scaffolds. Table 2 shows the hydrophilicity test results of PCL scaffolds with different mass fractions of 45s5, reflecting the hydrophilicity of the composites through the size of the water contact angle. These data reflect that in PCL/45s5 composite materials, with the increase of 45s5 bioglass content, the hydrophilicity of these materials increases. The water contact angle decreases from 94.03 ± 0.64° of pure PCL to 59.37 ± 1.10° of 20 wt% PCL/45s5, indicating a significant positive correlation between the water contact angle of PCL/45s5 composite materials and the content of 45s5 in the composite materials.

### 3.6. The Result of Rheology

**Figure 7:**
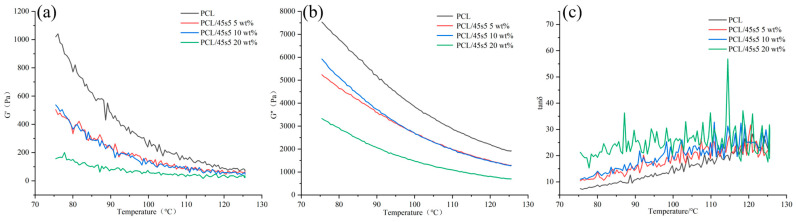
The result of rheology. (**a**) Energy storage modulus (G′); (**b**) loss modulus (G″); (**c**) loss tangent (tan δ).

Using a heating rheometer, we investigated the storage modulus G′ of composite materials in the temperature range of 75 °C to 125 °C, as shown in Figure 7a. The storage modulus G′ decreased with increasing temperature, indicating that the stiffness of the composite material decreased with increasing temperature, and this trend became more pronounced with increasing 45s5 bioglass content. The loss modulus G’, as shown in Figure 7b, shows that with the increase in 45s5 bioglass content, the loss modulus of the composite material decreases, indicating that the higher the 45s5 content, the lower the energy required for material deformation, resulting in a lower viscosity of the material. Figure 7c shows the ratio of storage modulus to loss modulus. In the experiment by Evangelos Daskalakis et al., the loss modulus and storage modulus of PCL, PCL/HA, PCL/TCP, and PCL/BIOGLASS all showed a decreasing trend with increasing temperature. This is a normal phenomenon. As the temperature increases, molecular motion within the sample intensifies, causing changes in the sample’s structure or chemical bonds, which in turn leads to increased loss and decreased storage modulus—a typical thermal effect [43].

### 3.7. Modulus of Elasticity

**Figure 8:**
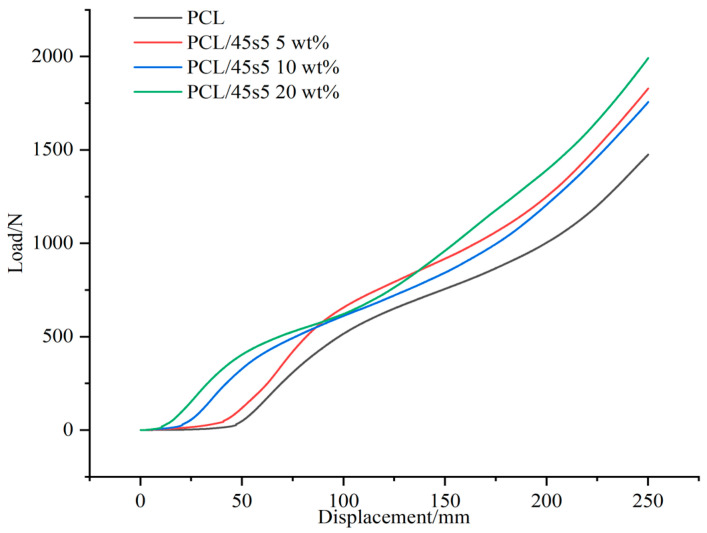
Elastic modulus of composite materials.

Using a universal material testing machine, our experiment investigated the elastic modulus of PCL and PCL/45s5 composite materials, including pure PCL and composite materials with 5 wt%, 10 wt%, and 20 wt% 45s5 content. The aim of this study is to investigate the impact of 45s5 on the mechanical properties of composite materials and to assess its potential as a bone-like material. Yet, the data presented in Figure 8 indicate that none of the composite materials satisfy the stringent mechanical specifications needed to effectively simulate cortical bone. The elastic modulus of cortical bone is influenced by several factors, including age, gender, degree of mineralization, and the direction of testing due to bone anisotropy. Generally, the elastic modulus of human cortical bone ranges from 10,000 N/mm to 20,000 N/mm [30]; the highest average elastic modulus observed in the sample is only 155.43 N/mm.

Although the introduction of 45s5 does indeed enhance the elastic modulus of composite materials, these changes are not sufficient to classify composite materials as suitable substitutes for cortical bone, indicating that there is a certain gap in the current use of composite materials in replacing bone formation.

### 3.8. In Vitro Biodegradation of Composite Scaffolds

Bone regeneration is a multifaceted biological process affected by numerous elements, such as bone classification, characteristics of the defect, personal age, overall health, nutritional condition, and vascular supply. To evaluate the biodegradability of bone scaffolds, we measured the weight loss rate of a 50 wt% porosity PCL/45s5 composite bone scaffold cultured in PBS simulated body fluid for 40 days. Table 3 shows the weight loss rate of the PCL scaffold over a period of 40 days. As the 45s5 bioglass content increases, the scaffold degrades more over the course of 40 days. This directly indicates that the integration of 45s5 greatly enhances the biodegradability of the PCL scaffold.

### 3.9. Antibacterial Experiment

**Figure 9:**
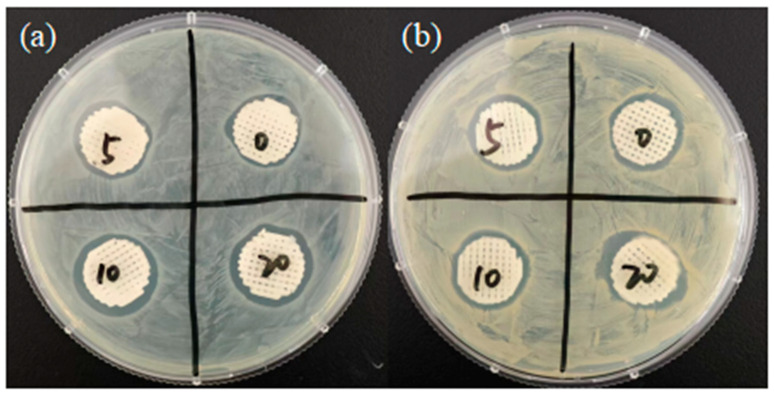
Antibacterial circle of composite scaffold. (**a**) *Escherichia coli*; (**b**) *Staphylococcus aureus*. 0 represents the pure PCL group, and 5, 10, 20 represent the experimental groups of three composite material scaffolds.

By observing the size of the antibacterial zone on LB plates coated with *Escherichia coli* and *Staphylococcus aureus*, the effect of the addition of 45s5 on the antibacterial properties of the scaffold was investigated, as shown in Figure 9 and Table 4. It was found that with the increase in 45s5 bioglass content in the composite material, the inhibitory ability of the scaffold against Gram-positive and Gram-negative bacteria showed an increasing trend.

The principle of Gram staining is to stain Gram-positive bacteria and Gram-negative bacteria with different colors based on the structure and composition of bacterial cell walls. During the staining process, the cell wall of Gram-positive bacteria can retain a purple staining agent, while Gram-negative bacteria cannot. Therefore, under the microscope, Gram-positive bacteria appear purple and Gram-negative bacteria appear pink.

**Figure 10:**
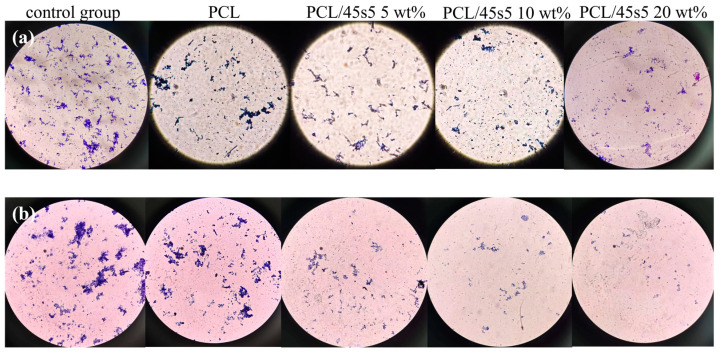
Antibacterial experiment Gram staining results. (**a**) *Escherichia coli*; (**b**) *Staphylococcus aureus*.

We obtained similar results through Gram staining. As shown in Figure 10, the inhibitory effect of the pure PCL group on Gram-positive bacteria and Gram-negative bacteria was similar to that of the control group without adding any antibacterial substances. With the increase in 45s5 bioglass content in the composite scaffold, the inhibitory effect of the composite scaffold on Gram-positive bacteria and Gram-negative bacteria was significantly improved.

## 4. Results and Discussion

This study constructed a porous PCL/45s5 composite scaffold using 3D printing technology and conducted physical and chemical properties, mechanical properties, in vitro degradation, and antibacterial testing. We attempted to construct a composite material bone defect filler through the above tests. With the increase in 45s5 bioglass, the scaffold’s capacity for regeneration is significantly improved. Bioactive glass is well known for its capacity to stick to living tissue, increase cellular activity, and encourage the formation of extracellular matrix components. Nevertheless, the mechanical strength of PCL/45s5 is far below the strength required for bone repair. As a result, it may not be suitable for load-bearing bone implants. Instead, it could be effectively used in the repair of non-load-bearing bone or cartilage.

According to the in vitro degradation rate results, the scaffold’s rate of degradation steadily rises as the amount of 45s5 in it increases. The breakdown rate and ion release rate of the scaffold can be controlled by altering the quantity of 45s5 bioglass, which could aid in managing the release of medications that are encapsulated. Furthermore, the compression tests of the composite scaffold samples demonstrate that the mechanical properties of the PCL/45s5 composite material are inadequate to satisfy the mechanical strength requirements for implanted load-bearing bones. However, it can be utilized to correct abnormalities in non-load-bearing bones or cartilage. The contact angle experiment indicates that the composite material’s hydrophilic surface facilitates cell adhesion and proliferation. Additionally, antibacterial experiments have shown that the inhibitory effect of PCL/45s5 composite scaffold on Gram-positive and Gram-negative bacteria tends to increase with the addition of 45s5 bioglass. However, further experimental verification is needed for its related performance after degradation and specific effects on cells.

## Figures and Tables

**Table 1:** Measurement results of weight, diameter, and height of composite scaffolds.

		1	2	3	Average	σ
PCL	Weight (g)	0.47	0.47	0.46	0.47	0.0058
	Diameter (mm)	14.92	15.24	14.98	15.05	0.1701
	Height (mm)	4.20	4.06	4.14	4.13	0.0702
PCL/45s5 5 wt%	Weight (g)	0.44	0.46	0.47	0.46	0.0153
	Diameter (mm)	15.16	15.62	15.36	15.38	0.2307
	Height (mm)	3.98	3.86	4.04	3.96	0.0917
PCL/45s5 10 wt%	Weight (g)	0.47	0.43	0.48	0.46	0.0265
	Diameter (mm)	15.78	14.98	15.52	15.43	0.4081
	Height (mm)	4.26	4.08	4.06	4.13	0.1102
PCL/45s5 20 wt%	Weight (g)	0.39	0.45	0.41	0.42	0.0306
	Diameter (mm)	15.20	15.74	15.36	15.43	0.2774
	Height (mm)	4.28	4.08	4.12	4.16	0.1058

**Table 2:** Contact angle test results.

	1	2	3	Average	σ
PCL	94.30	93.30	94.50	94.03	0.64
PCL/45s5 5 wt%	73.00	73.20	71.40	75.53	0.99
PCL/45s5 10 wt%	66.60	67.00	67.30	66.97	0.35
PCL/45s5 20 wt%	60.00	60.00	58.10	59.37	1.10

**Table 3:** The weight loss of the scaffolds.

	Before	After	Difference
PCL	0.4698 g	0.4680 g	0.0018 g
PCL/45s5 5 wt%	0.4472 g	0.4446 g	0.0026 g
PCL/45s5 10 wt%	0.4754 g	0.4687 g	0.0067 g
PCL/45s5 20 wt%	0.3980 g	0.3834 g	0.0146 g

**Table 4:** Antibacterial zone size.

	*Escherichia coli*				*Staphylococcus aureus*			
	PCL	5 wt% 45s5	10 wt% 45s5	20 wt% 45s5	PCL	5 wt% 45s5	10 wt% 45s5	20 wt% 45s5
Parallel 1 (cm)	1.95	1.92	1.99	1.98	1.81	1.82	1.90	2.35
	1.82	1.90	1.91	1.97	1.90	1.85	1.95	1.85
	1.90	1.91	1.98	1.95	1.86	1.88	1.97	1.90
Parallel 2 (cm)	1.82	1.87	2.08	2.09	1.70	1.90	1.95	1.98
	1.81	1.90	2.00	2.02	1.80	1.85	1.90	1.90
	1.88	1.85	2.02	2.01	1.82	1.80	1.88	1.91
Parallel 3 (cm)	1.91	1.85	1.92	1.91	1.86	1.90	1.90	1.97
	1.93	1.90	1.90	1.90	1.80	1.91	1.95	1.91
	1.90	1.82	1.95	1.91	1.85	1.92	1.90	1.90
Average (cm)	1.88	1.88	1.97	1.97	1.82	1.87	1.92	1.96

## Data Availability

All relevant data are within the manuscript.
